# Quality Indicators in Otolaryngology–Head and Neck Surgery: A Scoping Review

**DOI:** 10.1177/19160216251330627

**Published:** 2025-04-25

**Authors:** Phillip Staibano, Shireen Samargandy, Justin Cottrell, Lily Wang, Michael Au, Michael K. Gupta, Han Zhang, Doron D. Sommer, Christopher Walsh, Eric Monteiro

**Affiliations:** 1 Division of Otolaryngology–Head and Neck Surgery, Department of Surgery, McMaster University, Hamilton, ON, Canada; 2 Department of Health Methods, Evidence, and Impact, McMaster University, Hamilton, ON, Canada; 3 Department of Otolaryngology–Head and Neck Surgery, University of Toronto, Toronto, ON, Canada; 4 Mount Sinai Library Services, Sinai Health, Toronto, ON, Canada; 5 Department of Otolaryngology–Head and Neck Surgery, Sinai Health, University of Toronto, Toronto, ON, Canada

**Keywords:** quality indicators, otolaryngology, head and neck surgery, quality improvement, systematic review

## Abstract

**Importance:**

Quality indicators are used to evaluate the quality of healthcare delivery and as a speciality, otolaryngology–head and neck surgery (OHNS) is beginning to transition toward this empirical understanding of healthcare quality and delivery.

**Objective:**

To describe the number and quality of studies that have developed novel quality indicators for any subdiscipline in OHNS.

**Design:**

We performed a database search of MEDLINE (Ovid), EMBASE (Ovid), Web of Science, and Cochrane Database of Systematic Reviews. We did not employ language or study-type restrictions and included studies published from database inception to October 2024.

**Study Selection:**

Following abstract screening, 184 articles underwent full-text screen. Eligible studies developed quality indicators in any subdiscipline within OHNS. Article screening and full-text review was performed in duplicate.

**Data Extraction and Synthesis:**

We extracted study-specific and methodological data in duplicate. Quality appraisal was assessed using the Appraisal of Indicators through Research and Evaluation instrument.

**Results:**

We identified 10,592 studies, of which 25 studies developed new quality indicators. Quality indicator development studies primarily focused on otology/neurotology, pediatric OHNS, and head and neck surgery. Few studies investigated facial plastics, rhinology and skull base surgery, and laryngology. Most studies employed Delphi consensus methods and patient engagement was rare. Consensus methodology reporting was poor and indicators were often not validated. Outcome indicators were often measured with fewer studies investigation structure or process indicators.

**Conclusions:**

Quality indicators may help standardize and improve patient care in OHNS. Future research should focus on structure and process indicators, while improving reporting, optimizing panel composition, and validating quality indicators.

## Graphical abstract

**Graphical Abstract:**
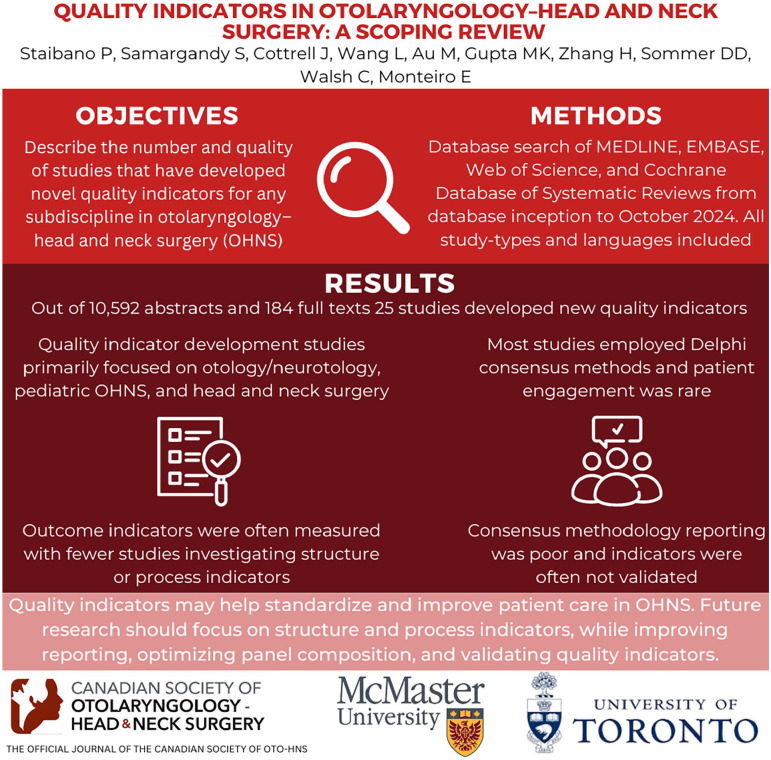

## Key Messages

• Quality indicator development research is ongoing in otolaryngology–head and neck surgery (OHNS)
• Most studies developed quality indicators in otology/neurotology, pediatric OHNS, and head and neck surgery with few studies investigating facial plastics, rhinology, and laryngology
• Optimizing consensus panel composition, improving reporting, and developing structure and process indicators will improve the impact of quality indicator research in OHNS

## Introduction

Healthcare systems are shifting toward an empirical understanding of patient care.^1,2^ Quality indicators are performance metrics that help standardize the evidence-based evaluation of healthcare quality and delivery, in addition to clinical outcomes.^3^ A common framework is the Donabedian model, which categorizes quality indicators into structure, process, and outcome measures (Table 1).^1^ An important and renowned application of surgical quality indicators is the American College of Surgeons National Surgical Quality Improvement Program (NSQIP), which is a quality indicator database connecting healthcare institutions and surgical disciplines across the United States.^4^ Since the beginning of this quality initiative, NSQIP-affiliated institutions have helped to improve clinical outcomes and healthcare delivery, for patients and healthcare practitioners.^4-6^

**Table 1.** Quality Indicators as Defined by the Donabedian Framework.

	Structure indicator	Process indicator	Outcome indicator
Definition	What is the context/setting in which healthcare is delivered to patients?	What actions are being undertaken to facilitate healthcare delivery to patients?	What is the outcome on patient health following healthcare delivery?
Examples	• Institution • Staff and equipment • Surgical volumes	• Preventive care • Diagnosis and treatment planning • Patient education	• Patient satisfaction • Morbidity and mortality • Health-related quality of life

Quality indicators are developed via a multistage process that requires analyzing peer-reviewed literature and clinical practice guidelines, while incorporating input from allied healthcare professionals, patients, and their families to generate target performance metrics for further scientific validation.^7,8^ An often-used development method for quality indicators involves a consensus approach, or Delphi method, whereby an expert panel of medical professionals establish candidate quality indicators that are then subject to successive rounds of discussion and selection until final quality indicators are identified for further validation.^9^ Over the past decade, researchers in otolaryngology–head and neck surgery (OHNS) have begun generating quality indicators to standardize and improve healthcare quality and delivery. So far, however, there has not yet been a fulsome review of the breadth of quality indicators in OHNS. Hence, the objective of this scoping review was to characterize and evaluate the quality of studies that have developed quality indicators in OHNS.

## Materials and Methods

This study was reported in accordance with the Preferred Reporting Items for a Systematic Review and Meta-Analysis (PRISMA) Scoping Review Extension Guidelines.^10^ As this is a scoping review, it did not require PROSPERO database registration.^11^

### Search Strategy

We reviewed all studies that reported quality indicators within OHNS in Medline, Embase, Web of Science, and Cochrane Database of Systematic Reviews from database inception to October 24, 2024, with the help of a librarian specialist (C.W.) (Appendix A). We performed a qualitative search of the reference lists of included articles and review articles. We did not include a language restriction. Further, a gray literature search of global professional organizations in OHNS and healthcare quality as well as gray literature databases was also performed (i.e., Google, Google Scholar, OAlster, OpenGrey). We searched the first 10 pages of all web page results.

### Study Eligibility

We included studies that addressed quality indicators in any subdiscipline in OHNS, including facial plastics and reconstructive surgery, general OHNS, head and neck surgery, laryngology, otology and neurotology, pediatric OHNS, and rhinology and skull base surgery. We defined development studies as those that utilized any consensus methods or other methodologies to identify quality indicators and/or outcome sets. We included adult and pediatric patients in any clinical setting. We did not include pathologies or clinical interventions involving the esophagus or thoracic cavity. We also did not include quality indicators in radiation or medical oncology for head and neck oncology.

We included peer-reviewed original research, systematic reviews, consensus and clinical guidelines, and guideline extraction articles that developed novel quality indicators in the context of clinical care. There was no language restriction. We also excluded any narrative reviews and editorials. We excluded any studies that proposed or evaluated existing quality indicators from the final descriptive analysis.

### Study Selection and Data Abstraction

**Figure 1.**
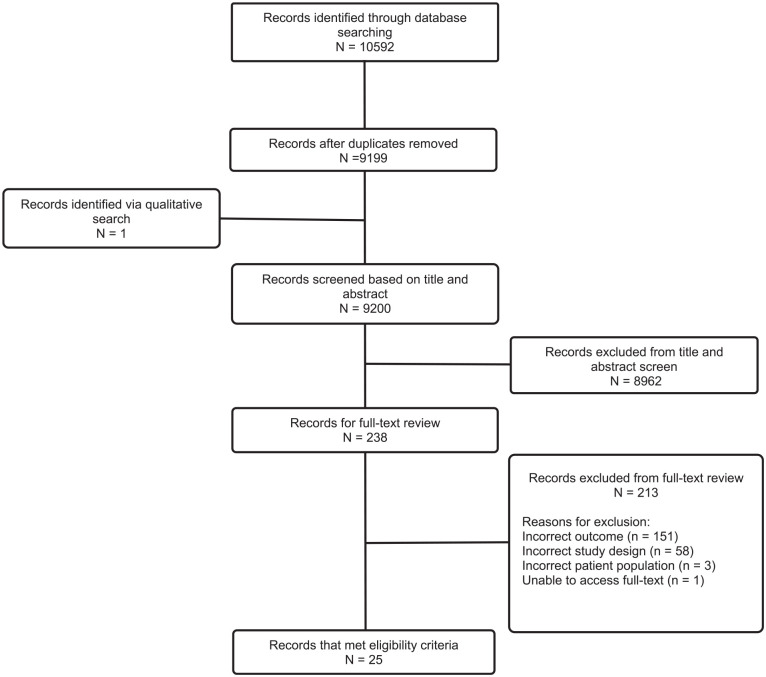
PRISMA flowchart for included studies. PRISMA, Preferred Reporting Items for a Systematic Review and Meta-Analysis.

Articles were reviewed following PRISMA guidelines (Figure 1). All review stages were completed by 2 reviewers (P.S., L.W.) and conflicts were resolved via discussion including 2 senior authors (S.S., J.C.). We utilized Covidence (Melbourne, Australia) for article screening. Data abstraction was performed by 3 authors (P.S., S.S., and L.W.), and all discrepancies were addressed by a senior reviewer (E.M.). We collected data pertaining to authorship, year of publication, study location, conflicts of interest, study design, methodology, as well as individual quality indicators and their associated details, including descriptor numerator and denominator.

### Quality Appraisal and Statistical Analysis

All included studies that developed novel quality indicators were evaluated using the validated Appraisal of Indicators through Research and Evaluation (AIRE) tool.^12^ The AIRE is a validated instrument that was designed to evaluate studies of quality indicators in healthcare.^13^ It comprised 20 items that address 4 quality domains: (1) purpose and organizational context, (2) stakeholder involvement, (3) scientific evidence, and (4) additional evidence. Each item is scored on a 4-point scale (i.e., 1: strongly disagree and 4: strongly agree). A standardized domain score was calculated: [(total score − minimum possible score)/(maximum score − minimum possible score) × 100%]. A score closer to 100% indicated higher methodological quality. Each domain was independently evaluated by 2 reviewers (P.S., L.W.) with discrepancies resolved by a third reviewer (S.S.). We analyzed all included studies via descriptive analyses, including measures of central tendency and standard deviation. We calculated frequencies and proportions for all categorical variables. All statistical analyses and tables were generated using Microsoft Excel (Ridgefield, WA, USA).

## Results

We identified 10,592 studies from the database search wherein 25 studies met eligibility criteria (Figure 1).^14-38^ All included studies developed new quality indicators in OHNS (Table 2). We found that most studies (n = 18, 72%) utilized Delphi, modified Delphi, or RAND-UCLA consensus methods. Candidate quality indicators were primarily selected from clinical practice guidelines and/or scientific literature (n = 21, 84%). These consensus studies primarily used an expert panel composed of medical specialists (n = 19, 76%) with few studies including patient or family engagement (n = 7, 28%). The most studied OHNS subdiscipline was pediatric OHNS (n = 7, 28%) with most studies evaluating otitis media or tonsillitis (Table 3). In otology and neurotology (n = 6, 24%), most studies evaluated tinnitus or vestibular disease. In head and neck surgery (n = 6, 24%), most studies evaluated head and neck cancer. Supplemental Table S1 describes the quality indicators selected in each included study.

**Table 2.** Summary of Quality Indicator Development Studies (N = 25).

Study characteristic	No. of studies (%)
Publication year	
2010—present	24 (96)
2000—2009	1 (4)
Study country	
Canada	8 (32)
USA	6 (24)
Netherlands	3 (12)
UK	2 (8)
Australia	2 (8)
Spain	2 (8)
Portugal	1 (4)
Brazil	1 (4)
Consensus methods	
Delphi/modified Delphi	13 (52)
RAND-UCLA appropriateness	7 (28)
Not reported	5 (20)
Expert panel composition	
Medical specialists only	17 (68)
Medical specialists and other healthcare professionals^a^	7 (28)
Not reported	1 (4)
Patient engagement	
No patient involvement reported	18 (72)
Patient and/or patient-centered organizations	7 (28)

Abbreviation: CPG, clinical practice guideline.

a Includes audiologists, nurses, speech-language pathologists, methodologists, psychologists, and administrators.

**Table 3.** Summary by Otolaryngology–Head and Neck Surgery Subdiscipline (N = 25).

Otolaryngology–head and neck surgery subdiscipline	No. of studies (%)
Pediatric OHNS	7 (28)
Otitis media^a^	3 (42.9)
Tonsillitis	2 (28.6)
Lymphatic malformations	1 (14.3)
Laryngeal anomalies	1 (14.3)
Otology and neurotology	6 (24)
Tinnitus/vestibular disorders	2 (33.3)
Hearing loss^b^	1 (16.7)
Cochlear implants	1 (16.7)
Cerumen impaction^c^	1 (16.7)
Cholesteatoma	1 (16.7)
Head and neck surgery	6 (24)
Head and neck cancer^d^	5 (83.3)
Primary hyperparathyroidism	1 (16.7)
Rhinology and skull base surgery	3 (12)
Chronic rhinosinusitis	2 (50)
Acute rhinosinusitis	1 (25)
General OHNS	2 (8)
General OHNS	1 (50)
Dysphagia	1 (50)
Facial plastics and reconstructive surgery	1 (4)
Rhinoplasty	1 (100)

Abbreviation: OHNS, otolaryngology–head and neck surgery.

a Includes acute otitis media, chronic suppurative otitis media, and otitis media with effusion.

b Includes sudden sensorineural hearing loss.

c Michel et al^30^ also investigated allergic rhinitis.

d Includes oral cavity, oropharyngeal, laryngeal, hypopharyngeal cancer, dysphagia in the context of head and neck cancer, and mandibular osteoradionecrosis.

### Study Quality Appraisal

All studies were evaluated using the AIRE instrument (n = 25; Supplemental Table S2). We found that the mean value for the *purpose* domain was 63.8% (range: 27%-87%). The primary deficiency in this domain was poor reporting of the criteria for selecting quality indicators. The mean value for the *stakeholder* domain *was* 54.5% (range: 5.5%-78%) and its primary deficiency was a lack of patient/family and allied health professional engagement. The mean value for the *scientific evidence* domain was 55.8% (range: 0%-89%) with reporting deficiency in the search methods and types of resources identified for quality indicator development. The mean value for the *additional evidence* domain was 41.8% (range: 15%-74%). These studies reported construct validity with adequate detail; however, we found limited reporting with regards to quality indicator description and measurement, test reliability, risk adjustment, and quality indicator piloting.

### Pediatric Otolaryngology–Head and Neck Surgery

There were 7 studies (28%) of pediatric OHNS that developed new quality indicators.^15,16,18,21,23,25,34^ Most studies investigated pediatric otitis media (Table 3).^21,23,34^ Four studies used a Delphi/modified Delphi consensus method, 2 used the RAND-UCLA method, and 1 study did not report the consensus method. Expert consensus panels included surgeons and physicians in all studies, while 2 studies also included allied health professionals (e.g., audiologists, nurses, speech language pathologies).^23,34^ One study incorporated patient and family surveys in developing outcome indicators for otitis media with effusion.^23^ Three studies extracted candidate indicators from peer-reviewed clinical practice guidelines, while all remaining studies drew candidate indicators from the peer-reviewed literature and expert opinion.^18,21,25^ Across all eligible studies, 113 novel quality indicators were developed using consensus methods: 72 (63.7%) were outcome indicators, 41 (36.3%) were process indicators, and none were structure indicators (Supplemental Table S1). Empirical validation was performed in one study.^25^

### Otology and Neurotology

There were 6 studies (24%) of otology and neurotology that developed new quality indicators (Table 3).^20,22,30,35,37,38^ Most studies evaluated tinnitus and Meniere’s disease. Two studies used the Delphi method while 2 studies used the RAND-UCLA method. Three studies (50%) extracted candidate measures from clinical practice guidelines. Two studies included patient input or health user stakeholders in their consensus panels.^22,35^ Three studies extracted candidate quality indicators from clinical practice guidelines.^20,30,38^ None of the studies performed validation of proposed indicators. Across all included studies, 41 novel quality indicators: 34 (82.9%) were outcome indicators, 7 (17.1%) were process indicators, and none were structure indicators (Supplemental Table S1).

### Head and Neck Surgery

There were 6 studies (24%) of head and neck surgery that developed novel quality indicators (Table 3).^24,27,29,32,33,36^ Five studies investigated head and neck cancer and one investigated primary hyperparathyroidism. Three studies (50%) used the modified RAND-UCLA appropriateness method, and 3 studies (50%) used Delphi or modified Delphi methods. Two studies employed patient-based organizations while remaining studies used expert panels that included both physicians and allied health professionals. All studies derived candidate indicators from published clinical guidelines, peer-reviewed studies, and expert opinion. No studies performed any empirical testing of selected quality indicators. Across all studies, 166 novel quality indicators were developed: 83 (50%) were outcome indicators, 77 (46.4%) were process indicators, and 6 (3.6%) were structure indicators.

### Rhinology and Skull Base Surgery

Three studies (12%) developed novel quality indicators in rhinology.^17,19,26^ These studies investigated acute bacterial and chronic rhinosinusitis. One study developed quality indicators for both cerumen impaction and allergic rhinitis.^30^ Two studies employed a RAND-UCLA method while the other study used a Delphi method. Three studies identified candidate quality indicators via clinical practice guidelines, while the other study identified candidate indicators via the peer-reviewed literature. Two studies described the expert panel to consist of physicians, while one study included patient engagement.^26^ A total of 38 quality indicators were developed with 20 (52.6%) being process indicators and 18 (47.4%) being outcome indicators. None of these studies performed quality indicator validation.

### Other Otolaryngology–Head and Neck Surgery Subdisciplines

Two studies investigated new quality indicators in general practice OHNS.^14,31^ These studies evaluated inpatient OHNS and dysphagia. One study used the Delphi consensus method.^14^ These authors also included allied health specialists and medical specialists within their expert consensus panel. Moraes et al describe 12 process outcomes related to dysphagia and swallowing assessment.^31^ Manahan et al identify candidate quality indicators from clinical practice guidelines, but do not describe a selection method.^28^ These authors identify 1 outcome and 3 process indicators. None of these studies performed quality indicator validation.

### Gray Literature

We identified 2 citations: (1) entnet.org, which is hosted by the American Academy of OHNS and (2) healthmonix.org, which is hosted by a private company involved with healthcare quality improvement. There were 49 quality indicators involving OHNS and general medicine, of which 47 were process indicators and 2 were outcome indicators. We did not identify any studies appraising these quality indicators.

## Discussion

With the success of the NSQIP, surgical disciplines continue to develop and validate quality indicators that connect healthcare networks and improve healthcare delivery.^8,39^ Herein, we present a scoping review aimed at describing all published OHNS quality indicators so that investigators can identify gaps in their knowledge and application. Schneider and Lavin identified several quality improvement databases and registries that are being used in OHNS to improve patient care and healthcare efficiencies.^40^ In our review, quality indicator studies primarily investigated patient management in otology, neurotology, pediatric OHNS, and head and neck surgery. These studies focused on otitis media, tinnitus and vestibular disorders, sudden and chronic hearing loss, tonsillitis, and head and neck cancer. Otology and neurotology are often limited by low-quality research and research volumes that make up less than half of all OHNS publications.^41,42^ There has also been a paucity of high-quality pediatric OHNS research published over the past 3 decades.^43^ Head and neck surgery is often studied in OHNS, but randomized trials in head and neck surgical oncology suffer from poor methodological quality.^44^ Core outcome sets are being developed to help standardize outcome assessment and reporting in head and neck surgery.^45^ Minimal quality indicator development research has been performed in facial plastics, rhinology and skull base surgery, and laryngology. A drive toward an evidence-based understanding and application of quality indicators will contribute to clinical research and standardization of healthcare quality in all subdisciplines of OHNS.

Most studies employed the Donabedian framework, wherein quality indicators were categorized as structure, process, and outcome measures. The Donabedian framework has been validated in other surgical specialities and may help facilitate the implementation of eHealth into healthcare systems.^46-48^ The Institute of Medicine described 6 domains of healthcare quality: patient safety, effectiveness, patient-centeredness, timeliness, efficiency, and equity that have been applied to medical populations, but we did not find any consistent use of this framework and it has been less applied to surgical patients.^49^ When applying the Donabedian framework to OHNS quality indicators, we found that structure, process, and outcome indicators were all included, but most studies developed only process and outcome indicators. Similarly in vascular surgery, structure and process indicators were rarely studied when compared to outcome indicators.^50^ In a review of surgical oncology studies, however, process indicators were studied more often than both structure and outcome indicators.^51^ In other non-OHNS surgical specialties, process indicators have shown to be better drivers of hospital quality improvement when compared to outcome indicators.^50,52^ Further, Rademakers et al suggested that structure and process indicators were more likely to improve patient ratings of quality of care.^53^ Hence, structure and process indicators are important for internal quality improvement, while outcome indicators help inform the public about institutional differences in healthcare.^54^ As such, we suggest that in addition to outcome indicators, future studies focus on structure and process indicators as these are less often studied in OHNS but contribute to evidence-based healthcare quality improvement initiatives.

At present, there are no standardized methodologies or guidelines for conducting consensus research for developing quality indicators.^55-57^ In our review, we found that a Delphi or modified Delphi method was most often employed by authors, but reporting detail was often lacking, which is consistent with the literature.^8^ Further, consensus panels primarily included physicians but often did not gather the opinions of other stakeholder, such as administrators, allied health professionals, patients, and families, which is consistent with other studies.^58^ In addition, quality indicators were primarily refined by local experts from regional hospital networks, but often did not incorporate opinions from other countries. We also found that quality indicators were not piloted or validated in these studies, nor were they provided to quality improvement entities (i.e., Agency for Healthcare Research and Quality) for feedback. Validating healthcare quality indicators is important as it prevents improper data entry and bias by providers while ensuring the chosen indicators are reflective of healthcare quality.^59,60^ In evaluating these studies using the AIRE instrument, deficiencies existed in the lack of patient engagement and allied health professionals within the expert panel, poor reporting of consensus methodologies and methods used to identify candidate quality indicators, and a lack of empirical piloting or risk adjustment for chosen quality indicators. We also recommend that investigators consider developing reporting guidelines for consensus methods used to develop quality indicators. Advances in the standardization of electronic medical record management across North America may help to drive empirical piloting and validation of quality indicators. Moreover, future studies may benefit from evaluating and developing quality indicators for marginalized communities and patients in OHNS, such as head and neck cancer patients, who require multidisciplinary care.^61,62^

There are several limitations of this study. First, we did not empirically evaluate or validate these quality indicators. Second, we employed broad library search parameters using terms relevant to OHNS, but these terms may not have captured quality indicators related to allied health or patient engagement in OHNS.

Quality indicators are being developed and tested to improve healthcare quality and delivery in OHNS. Consensus methods for identifying quality indicators are gaining popularity, but investigators must improve study reporting, patient and relevant stakeholder engagement, and perform empirical validation of novel quality indicators. We should also consider focusing on structure and process indicators since they are less studied but may better inform quality improvement initiatives when compared to outcome indicators. As we proceed toward an era of eHealth and personalized medicine, a better understanding of quality indicators in OHNS may help to improve healthcare standardization, institutional accountability, and professional collaboration around the world.

## Supplemental Material

sj-docx-1-ohn-10.1177_19160216251330627 – Supplemental material for Quality Indicators in Otolaryngology–Head and Neck Surgery: A Scoping Review Supplemental material, sj-docx-1-ohn-10.1177_19160216251330627 for Quality Indicators in Otolaryngology–Head and Neck Surgery: A Scoping Review by Phillip Staibano, Shireen Samargandy, Justin Cottrell, Lily Wang, Michael Au, Michael K. Gupta, Han Zhang, Doron D. Sommer, Christopher Walsh and Eric Monteiro in Journal of Otolaryngology - Head & Neck Surgery

sj-docx-2-ohn-10.1177_19160216251330627 – Supplemental material for Quality Indicators in Otolaryngology–Head and Neck Surgery: A Scoping Review Supplemental material, sj-docx-2-ohn-10.1177_19160216251330627 for Quality Indicators in Otolaryngology–Head and Neck Surgery: A Scoping Review by Phillip Staibano, Shireen Samargandy, Justin Cottrell, Lily Wang, Michael Au, Michael K. Gupta, Han Zhang, Doron D. Sommer, Christopher Walsh and Eric Monteiro in Journal of Otolaryngology - Head & Neck Surgery

## Appendix A: Search Strategy

Database: Ovid MEDLINE(R) and Epub Ahead of Print, In-Process & Other Non-Indexed Citations, Daily and Versions(R) <1946 to January 20, 2020> Search Strategy:

1 exp Otolaryngology/ (12873)
2 otorhinolaryngologic surgical procedures/ or adenoidectomy/ or laryngectomy/ or laryngoplasty/ or laryngoscopy/ or
 nasal surgical procedures/ or rhinoplasty/ or neck dissection/ or otologic surgical procedures/ or cochlear implantation/ or mastoidectomy/ or middle ear ventilation/ or myringoplasty/ or ossicular replacement/ or stapes surgery/ or stapes mobilization/ or tympanoplasty/ or pharyngectomy/ or tonsillectomy/ or tracheostomy/ or tracheotomy/
 (87847)
3 “head and neck neoplasms”/ or “squamous cell carcinoma of head and neck”/ or *facial neoplasms/ or mouth
 neoplasms/ or gingival neoplasms/ or leukoplakia, oral/ or leukoplakia, hairy/ or lip neoplasms/ or palatal neoplasms/ or salivary gland neoplasms/ or parotid neoplasms/ or sublingual gland neoplasms/ or submandibular gland neoplasms/ or tongue neoplasms/ or otorhinolaryngologic neoplasms/ or ear neoplasms/ or laryngeal neoplasms/ or nose neoplasms/ or paranasal sinus neoplasms/ or maxillary sinus neoplasms/ or pharyngeal neoplasms/ or hypopharyngeal neoplasms/ or nasopharyngeal neoplasms/ or nasopharyngeal carcinoma/ or oropharyngeal neoplasms/ or tonsillar neoplasms/ or parathyroid neoplasms/ or thyroid neoplasms/ or thyroid cancer, papillary/ or thyroid nodule/ (248670)
4 *otorhinolaryngologic diseases/ or ear diseases/ or cholesteatoma, middle ear/ or ear deformities, acquired/ or
 ear neoplasms/ or earache/ or hearing disorders/ or hearing loss/ or deafness/ or hearing loss, bilateral/ or hearing loss, conductive/ or hearing loss, functional/ or hearing loss, high-frequency/ or hearing loss, mixed conductive-sensorineural/ or hearing loss, sensorineural/ or hearing loss, sudden/ or hearing loss, unilateral/ or tinnitus/ or labyrinth diseases/ or cochlear diseases/ or endolymphatic hydrops/ or meniere disease/ or labyrinthitis/ or vestibular diseases/ or bilateral vestibulopathy/ or vertigo/ or benign paroxysmal positional vertigo/ or myringosclerosis/ or otitis/ or otitis externa/ or otitis media/ or mastoiditis/ or otitis media with effusion/ or otitis media, suppurative/ or petrositis/ or otomycosis/ or otosclerosis/ or retrocochlear diseases/ or auditory diseases, central/ or auditory perceptual disorders/ or hearing loss, central/ or vestibulocochlear nerve diseases/ or “*neuroma, acoustic”/ or vestibular neuronitis/ or vestibulocochlear nerve injuries/ or tympanic membrane perforation/ or laryngeal diseases/ or granuloma, laryngeal/ or laryngeal edema/ or laryngeal neoplasms/ or laryngeal nerve injuries/ or recurrent laryngeal nerve injuries/ or laryngitis/ or croup/ or laryngocele/ or laryngomalacia/ or laryngostenosis/ or supraglottitis/ or tuberculosis, laryngeal/ or vocal cord dysfunction/ or vocal cord paralysis/ or voice disorders/ or exp nose diseases/ or exp otorhinolaryngologic neoplasms/ or exp pharyngeal diseases/ (351736)
5 thyroid neoplasms/ or thyroid cancer, papillary/ or thyroid nodule/ (51552)
6 1 or 2 or 3 or 4 or 5 (571070)
7 *“quality of health care”/ or guideline adherence/ or “outcome and process assessment (health care)”/ or “outcome
 assessment (health care)”/ or *patient outcome assessment/ or patient reported outcome measures/ or *treatment outcome/ or *progression-free survival/ or “process assessment (health care)”/ or *quality assurance, health care/ or total quality management/ or quality improvement/ or “meaningful use”/ or value-based health insurance/ or quality indicators, health care/ or risk adjustment/ or “standard of care”/ or benchmarking/ or “*guidelines as topic”/ or *“standard of care”/ (254458)
8 6 and 7 (3813)
9 quality indicator*.ti. (2095)
10 (process adj1 (measur* or indicator*)).ti. (301)
11 (structure adj1 (measure* or indicator*)).ti. (111)
12 (outcome* adj1 (measure* or indicator*)).ti. (5941)
13 (candidate* adj1 (indicator* or measure*)).ti. (12)
14 QI.ti. (1320)
15 QIs.ti. (12)
16 7 or 9 or 10 or 11 or 12 or 13 or 14 or 15 (258855)
17 otolaryngology.ti,ab,kw. (9256)
18 (otorhinolaryngologic surgical procedures or adenoidectomy or laryngectomy or laryngoplasty or laryngoscopy or
 nasal surgical procedures or rhinoplasty or neck dissection or otologic surgical procedures or cochlear implantation or mastoidectomy or middle ear ventilation or myringoplasty or ossicular replacement or stapes surgery or stapes mobilization or tympanoplasty or pharyngectomy or tonsillectomy or tracheostomy or tracheotomy).ti,ab,kw. (68446)
19 (((head and neck) or facial or mouth or gingival or lip or palatal or salivary gland or parotid or sublingual
 gland or submandibular gland or tongue or otorhinolaryngologic or ear or laryngeal or nose or paranasal sinus or maxillary sinus or pharyngeal or hypopharyngeal or nasopharyngeal or nasopharyngeal or oropharyngeal or tonsillar or parathyroid or thyroid) adj1 (cancer* or tumor* or tumour* or carcinoma* or malignan* or neoplasm*)).ti,ab,kw. (118665)
20 ((squamous cell carcinoma of head and neck) or leukoplakia or thyroid cancer papillary or thyroid
 nodule).ti,ab,kw. (8583)
21 (otorhinolaryngologic disease* or ear disease* or cholesteatoma or ear deform* or middle ear deform* or earache
 or hearing disorder* or hearing loss or deafness or hearing loss or bilateral hearing loss or conductive hearing loss or functional hearing loss or high frequency hearing loss or mixed conductive sensorineural hearing loss or sensorineural hearing loss or sudden hearing loss or unilateral hearing loss or tinnitus or labyrinth disease* or cochlear disease* or endolymphatic hydrops or meniere disease or labyrinthitis or vestibular diseases or bilateral vestibulopathy or vertigo or benign paroxysmal positional vertigo or myringosclerosis or otitis or otitis externa or otitis media or mastoiditis or otitis media with effusion or suppurative otitis media or petrositis or otomycosis or otosclerosis or retrocochlear
 disease* or central auditory disease* or auditory perceptual disorder* or central hearing loss or vestibulocochlear nerve disease* or acoustic neuroma or vestibular neuronitis or vestibulocochlear nerve injur* or tympanic membrane perforation or laryngeal disease* or laryngeal granuloma or laryngeal edema or pharyngeal disease* or laryngeal nerve
 injur* or recurrent laryngeal nerve injur* or laryngitis or croup or laryngocele or laryngomalacia or laryngostenosis or supraglottitis or laryngeal tuberculosis or vocal cord dysfunction or vocal cord paralysis or voice disorders or nose disease*).ti,ab,kw. (128463)
22 ((otorhinolaryngologic or ear or laryngeal) adj1 (cancer* or tumor* or tumour* or carcinoma* or malignan* or
 neoplasm*)).ti,ab,kw. (10261)
23 ((thyroid or thyroid papillary or thyroid nodule) adj1 (cancer* or tumor* or tumour* or carcinoma* or malignan*
 or neoplasm*)).ti,ab,kw. (42319)
24 17 or 18 or 19 or 20 or 21 or 22 or 23 (310656)
25 6 or 24 (652997)
26 16 and 25 (4409)
27 exp animals/ not humans/ (4665685)
28 26 not 27 (4399)
