# Supplementary material for: Quality Indicators in Otolaryngology–Head and Neck Surgery: A Scoping Review
Source: J Otolaryngol Head Neck Surg. 2025 Apr 25;54:19160216251330627. doi: 10.1177/19160216251330627 (PMC12035303; doi:10.1177/19160216251330627)
Supplement: sj-docx-1-ohn-10.1177_19160216251330627 – Supplemental material for Quality Indicators in Otolaryngology–Head and Neck Surgery: A Scoping Review [file sj-docx-1-ohn-10.1177_19160216251330627.docx]

| **Table S1.** Study quality appraisal of quality indicator development studies (N = 25) | | | | | | | | |
| --- | --- | --- | --- | --- | --- | --- | --- | --- |
| **Author** | **Year** | **Country** | **Clinical details** | **Consensus method/study design** | **Quality indicators** | | | |
|  |  |  |  |  | **Structure** | **Process** | **Outcome** | **Other** |
| Head and neck surgery | | | | | | | | |
| 1. Hernandez et al. | 2024 (24) | Spain | Head and neck cancer | Delphi | 1. Access to an anatomic pathology service for immunohistochemistry  2. Access to a biomarker evaluation service  3. Existence of a multidisciplinary Tumour Board | 1. Adequate imaging study prior to initiating treatment  2. Whole-body PET/CT in tumours of unknown primary to determine the therapeutic strategy  3. Histopathological study prior to initiating treatment  4. Complete staging with TNM system prior to initiating treatment  5. Routine evaluation of Epstein–Barr virus and human papillomavirus  6. Multidisciplinary assessment prior to initiating treatment  7. Assessment of patients’ nutritional status prior to initiating treatment  8. Adequate oral cavity and dental assessment by an expert prior to initiating radiotherapy  9. Initiate treatment with curative intent within 14 days of the therapeutic decision  10. Complete tumour resection in patients undergoing surgery with curative intent  11. Initiate treatment with adjuvant radiotherapy within 6 weeks of surgery  12. Use of intensity-modulated radiotherapy in radical radiotherapy  13. Adequate use of monotherapy in the early stages of the disease  14. Adjuvant chemoradiotherapy with cisplatin in patients with stage III or IV HNSCC  15. Adequate indication for chemoradiotherapy in patients with locally advanced disease  16. Determination of PD-L1 expression  17. Access to immunotherapy by eligible patients with recurrent and/or metastatic disease  18. Tumour board assessment of patients with local or systemic recurrence  19. Assessment of second- or third-line therapy in patients with recurrent and/or meta-static head and neck cancer  20. Proposal of participation in clinical trials  21. Adequate follow-up after treatment completion  22. Evaluation of thyroid function after neck irradiation | 1. Assessment of response to chemoradiotherapy after completing radical treatment in patients with locally advanced (stage III, IVA and IVB)  Disease  2. Assessment of response to chemoradiotherapy in locally advanced patients  (stage III, IVA and IVB) 8–12 weeks after radical treatment completion  3. Mortality after surgery  4. Mortality after non-surgical treatment with radical intent | None |
| 2. Joaquim et al. | 2023 (27) | Portugal | Head and neck cancer | Delphi | None | None | 49 outcome indicators, but unable to access from article appendix | None |
| 3. Manduchi et al. | 2024 (29) | Canada | Dysphagia in head and neck cancer | Delphi | None | None | 1. Difficulty swallowing  2. Difficulty swallowing liquids  3. Coughing when swallowing  4. Choking when swallowing  5. Pain when swallowing  6. Difficulty maintaining weight due to swallowing difficulty  7. Difficulty swallowing impacting quality of life | None |
| 4. Noltes et al. | 2022 (32) | Canada | Primary hyperparathyroidism | RAND-UCLA | None | 1. Primary hyperparathyroidism is diagnosed on biochemical grounds, and biochemical evaluation of suspected pHPT should include adequate calcium measurement (total calcium, corrected calcium, ionized calcium as appropriate), PTH, creatinine, and 25-hydroxyvitamin D levels  2. 24-hour urinary calcium and vitamin D serum levels should be measured in patients with pHPT, specifically those at risk for familial hypocalciuric hypercalcemia  3. Parathyroidectomy is indicated, and is the preferred treatment, for all patients with symptomatic pHPT  4. Parathyroidectomy is indicated regardless of whether objective symptoms are present or absent when the serum calcium level is >1 mg/dL above normal  5. Parathyroidectomy is indicated regardless of whether objective symptoms are present or absent in patients with osteoporosis, fragility fracture, or evidence of vertebral compression fracture on spine imaging  6. Parathyroidectomy is indicated regardless of whether objective symptoms are present or absent when there is objective evidence of kidney involvement, including silent nephrolithiasis on kidney imaging, nephrocalcinosis, hypercalciuria with increased stone risk, or impaired kidney function  7. Parathyroidectomy is indicated regardless of whether objective symptoms are present or absent when pHPT is diagnosed at 50 years or younger  8. Preoperative imaging should start with cervical ultrasonography combined with sestamibi or 4-dimensional computed tomography  9. Patients should not receive medical therapy (cinacalcet) for definitive management  10. If the patient meets an indication for parathyroidectomy, negative imaging results should not preclude parathyroidectomy  11. In patients with asymptomatic pHPT, abdominal imaging should be performed for detection of nephrocalcinosis or nephrolithiasis because both are indications for parathyroidectomy  12. Dual-energy x-ray absorptiometry should be performed for all patients with pHPT  13. Appropriate surgical strategies for parathyroidectomy include limited parathyroid exploration with use of intraoperative PTH and four gland parathyroid exploration | 1. Patients undergoing surgery for pHPT should have a cure rate approaching 98%  2. Persistent hyperparathyroidism rate  3. Postoperative complication rate: recurrent laryngeal nerve paralysis and cervical hematoma  4. Length of stay  5. Re-operative parathyroidectomy rate | None |
| 5. Ouwens et al. | 2007 (33) | Netherlands | Head and neck cancer | Not described | None | 1. No. of patients who know who to talk to for information and questions  2. Availability of an information protocol  3. No. of patients who were well-informed on all information items  4. No. of patients who said they were offered emotional support  5. No. of patients who were informed about the possibilities to contact companions in distress  6. Availability of a multidisciplinary alcohol abstinence protocol  7. No. of patients who had been asked about alcohol use  8. No. of patients with alcohol problems who were offered support  9. Availability of a multidisciplinary stop-smoking protocol  10. No. of patients who had been asked about smoking behaviour  11. No. of smokers who were offered support to stop smoking  12. No. of patients who could see a specialist one day after referral  13. No. of patients who had all necessary diagnostic procedures on day of their first visit to the specialist  14. No. of patients who started their first treatment within thirty days after their first visit to the specialist: operation, radiotherapy, chemotherapy  15. No. of patients who said that transition went seamlessly: to the head and neck centre, within the hospital between departments, from the head and neck centre returning home  16. No. of patients with swallowing problems after leaving the hospital who were offered arrangements about follow-up  17. No. of patients who had a radical neck dissection or radiation in this area and with whom arrangements were made about follow-up regarding their speech revalidation  18. No. of patients who were monitored regarding their nutrition health status before, during, and after their treatment 19. Availability of a radiologist who had experience with patients with HNC  20. No. of assessments of CT and MRI procedures by this radiologist 21. No. of patients with cancer of the mouth or oropharynx who had been seen by a maxillofacial expert team  22. No. of times clinicians had contact by telephone with the pathologist in case of a negative biopsy with suspicion of malignancy  23. No. of times all results of diagnostic procedures performed earlier were available during the patient’s first clinical visit | None | None |
| 6. Van Overveld et al. | 2017 (36) | Netherlands | Head and neck cancer | Delphi | 1. Accessibility of psychological screening scores  2. Presence of case manager/oncology nurse  3. Assignment of nurse to every 100 new head and neck cancer patients | *Dietician*  1. Malnutrition screening  2. Malnutrition monitoring  3. Referral to dietician  *Psychologist*  4. Psychological screening  5. Psychological screening results in patient records  6. Relevance of psychology referral discussed with patient  *Oral hygienist*  7. Fluoride caps for prevention of caries due to RT  8. Use of salt/soda solution  9. Scattering caps for prevention of mucositis  *Physiotherapist (PT) or SLP*  10. Control of movement  11. Referral to PT by physician  12. Structured medical transfer or follow-up treatment when at high risk of shoulder disability  13. Rehabilitation of swallowing, speech, and/or voice complaints  14. Use of swallow screening, fibre-optic endoscopic evaluation of swallowing and/or video fluoroscopy  15. Follow-up on swallowing, speech, and/or voice complaints  *Other*  16. Information about effects of alcohol and smoking  17. Presence of case manager/NP at conversation to discuss prognosis  18. Structured medical transfer to external allied health professionals and GP  19. Presence of case manager/NP during multidisciplinary team meeting (MTM)  *Medical team*  20. Pathological status of tumour  21. Presence of practitioner who is responsible for the patient in the MTM  22. MTM takes place before treatment  23. Presence of other disciplines in the multidisciplinary team meeting  24. Treatment plan available  25. Conditions for treatment plan  26. Involvement of dental team when treated with radiotherapy  27. Involvement of PT when treatment involves neck dissection  28. Control of thyroid function  29. Referral to hospital  30. Finishing diagnostics  31. Start first treatment  32. Start second treatment | 1. Healthcare status of the patient  2. Tumour recurrence  3. Complications  4. Quality of life  5. Patient experience | None |
| Otology and neurotology | | | | | | | | |
| 1. Cottrell et al. | 2021 (20) | Canada | Meniere’s disease | RAND-UCLA | None | 1. Sensorineural hearing loss (SNHL) must be documented audiometrically in the treated ear on at least one occasion to permit the diagnosis of Meniere’s disease  2. Intratympanic dexamethasone can be offered as a treatment option for patients with Meniere’s disease and intractable vertigo  3. Intratympanic gentamicin injection may help control Meniere episodes and should be considered as initial destructive therapy  4. Labyrinthectomy or vestibular neurectomy can be considered in patients failing intratympanic gentamicin therapy  5. Clinicians should not routinely order electrocochleography to establish the diagnosis of Meniere’s disease | None | None |
| 2. Cottrell et al. | 2021 (38) | Canada | Sudden sensorineural hearing loss | RAND-UCLA | None  None | 1. Distinguish between conductive and sensorineural hearing loss in a patient presenting with sudden hearing loss  2. Identify presumptive sudden sensorineural hearing loss for bilateral sudden hearing loss, recurrent episodes of sudden hearing loss, or focal neurological findings  3. Clinician should not order CT of the head/brain in the initial evaluation of a patient with presumptive sudden sensorineural hearing loss  4. Time to first audiogram  5. Clinician should not obtain routine laboratory testing for patients with idiopathic sudden sensorineural hearing loss (ISSNHL)  6. Clinicians should not routinely prescribe antivirals, thrombolytics, vasodilators, or vasoactive substances to patients with ISSNHL  7. Clinicians should obtain audiometric evaluation within 6 months of diagnosis for patients with ISSNHL  8. Clinicians should counsel patients with incomplete recovery of hearing about the possible benefits of amplification and HAT and other supportive measure  9. Clinicians should evaluate patients with ISSNHL for retrocochlear pathology by obtaining an MRI  10. Time to referral  11. Time to treatment | None  None | None |
| 3. Hall et al. | 2018 (22) | UK | Tinnitus | Delphi | None | None | 1. Ability to ignore  2. Concentration  3. Mood  4. Negative thoughts and beliefs  5. Sense of control  6. Tinnitus acceptance  7. Tinnitus loudness  8. Tinnitus intrusiveness  9. Quality of sleep | None |
| 4. Michel et al. | 2022 (30) | USA | Cerumen impaction | Not described | None | 1. Manual removal  2. Hearing aids and otoscopy  3. Treatment | None | None |
| 5. ten Tije et al. | 2020 (35) | Netherlands | Cholesteatoma | Delphi | None | None | 1. The presence or absence of a cholesteatoma in the first 5 years after surgical removal of cholesteatoma  2. Hearing level after surgical removal of cholesteatoma  3. The documented assessment of patient’s complaints with a validated patient reported outcome measures questionnaire | None |
| 6. Vila et al. | 2016 (37) | USA | Cochlear implantation | Modified Delphi | None | 1. Preoperative vaccination  2. Immediate facial nerve preservation  3. Delayed facial nerve preservation | 1. Postoperative infection rate  2. Reoperation rate  3. Speech perception  4. Cochlear implant usage  5. Quality of life improvement after implantation | None |
| Pediatric otolaryngology–head and neck surgery | | | | | | | | |
| 1. Balakrishnan et al. | 2015 (15) | USA | Head and neck lymphatic malformations | Modified Delphi | None | None | 1. Ability to communicate verbally related to lymphatic malformation  2. Age of treatment start  3. Airway compromise  4. Cranial nerve injury  5. Death  6. Degree of response  7. Feeding problems  8. Infection  9. Lymphatic malformation composition  10. Lymphatic malformation volume  11. Lymphatic malformation stage  12. Macroglossia  13. Measures of functional status  14. Measure of quality of life  15. Number and duration of lymphatic malformation  -related admissions  16. Number of interventions within a certain time frame  17. Need for tracheostomy  18. Pain  19. Patient/parent discomfort  20. Recurrent swelling  21. Skin involvement  22. Skin necrosis  23. Subsite(s) affected  24. Subsite specific metrics  25. Treatment goals  26. Treatment goals achieved  27. Unplanned hospitalizations |  |
| 2. Balakrishnan et al. | 2018 (16) | USA | Laryngotracheal reconstruction | Modified Delphi | None | None | 1. Long-term patency (10-year)  2. Mortality  3. Need for revision  4. Need for adjunctive procedures  5. Number of subsequent open procedures  6. Number of subsequent endoscopic procedures  7. Number of dilations  8. Parent/caregiver satisfaction  9. Patient satisfaction  10. Quality of life | None |
| 3. Cottrell et al. | 2020 (18) | Canada | Tonsillitis | RAND-UCLA | None | 1. Swabbing the throat and testing for GAS pharyngitis by RADT and/or culture should be performed  2. In children and adolescents, a negative RADT test should be backed up by a throat culture  3. In children and adolescents, a positive RADT test should not be backed up by a throat culture  4. Anti-streptococcal antibody titers are not recommended in the routine diagnosis of acute pharyngitis as they reflect past but not current events  5. After appropriately treating acute streptococcal tonsillitis, routine blood tests or urine examinations or cardiologic diagnostics such as ECG are not indicated  6. Patients with acute GAS pharyngitis should be treated with an appropriate antibiotic  7. Adjunctive therapy with a corticosteroid is not recommended in the management of GAS  Pharyngitis  8. Clinicians may recommend tonsillectomy for recurrent throat infection meeting appropriate criteria  9. Clinicians should administer a single intravenous dexamethasone to children undergoing tonsillectomy  10. Clinicians should not administer or prescribe perioperative antibiotics to children undergoing tonsillectomy  11. Clinicians should advocate for pain management after tonsillectomy and educate caregivers about the importance of managing and reassessing pain  12. Clinicians who perform tonsillectomy should determine their rate of primary and secondary post tonsillectomy hemorrhage at least annually  13. Clinicians should arrange for overnight, inpatient monitoring of children after tonsillectomy if they are <3 years old or have severe obstructive sleep apnea  14. Clinicians should recommend ibuprofen, acetaminophen, or both for pain control after tonsillectomy  15. Clinicians must not administer or prescribe codeine, or any medication containing codeine, after tonsillectomy in children younger than 12 years | 1. Rate of post-operative tonsillectomy readmissions | None |
| 4. Cottrell et al. | 2024 (21) | Canada | Acute otitis media (AOM) | UCLA-RAND | None | 1. The clinician should prescribe antibiotic therapy for AOM  (bilateral or unilateral) in children 6 months and older with severe signs or symptoms  2. Patients diagnosed with AOM and being observed with close  follow-up should receive antibiotic therapy if symptoms worsen or fail to improve within 72 h of symptom onset  3. Clinicians should prescribe amoxicillin for AOM when a decision  to treat with antibiotics has been made  4. In the perioperative period, clinicians should educate caregivers  of children with tympanostomy tubes regarding the expected duration of tube function, recommended follow-up schedule,  and detection of complications  5. Clinicians should prescribe systemic antimicrobial therapy for patients with complicated AOM at presentation  6. Clinicians should provide a referral for otolaryngology opinion  for patients with complicated AOM at presentation  7. Clinicians should prescribe topical antibiotic eardrops only,  without oral antibiotics, for children with uncomplicated acute  tympanostomy tube otorrhea  8. Persistent middle ear effusion after AOM does not require antimicrobial therapy | None | None |
| 5. Harman et al. | 2015 (23) | USA | Otitis media with effusion in cleft lip and palate | Delphi | None | None | 1. Acute otitis media  2. Cholesteatoma  3. Chronic otitis media  4. Hearing  5. Listening skills  6. Otalgia  7. Otitis media with effusion  8. Receptive language skills  9. Psychosocial development  10. Speech development  11. Side effects of treatment | None |
| 6. Hibbert et al. | 2019 (25) | Australia | Tonsillitis | Modified UCLA-RAND / patient survey | None | 1. Children aged 3-14 y with a sore throat had temperature assessed 2. Children with a sore throat and with no other symptoms or signs of tonsillitis were not described antibiotics  3. Parents of children with a sore throat were instructed to provide fluids  4. Children aged <4 y with a sore throat and associated cough who did not require hospitalization were not prescribed antibiotics  5. Children aged 3-14 y assessed as high risk or GABHS positive and allergic to penicillin were prescribed oral erythromycin  6. Children with recurrent acute sore throat with episodes that were disabling and prevented normal functioning were indicated for tonsillectomy  7. Children who had a tonsillectomy and adenoidectomy were not administered perioperative antibiotics  8. Children who had a tonsillectomy and adenoidectomy were given a stat dose of dexamethasone  9. Children who had a tonsillectomy and adenoidectomy were prescribed antiemetic medication post-surgery  10. Children who had a tonsillectomy and adenoidectomy were informed of the potential for pain to increase for up to 6 days post-surgery  11. Parents/carers of children who had a tonsillectomy and adenoidectomy were informed of the risk of postoperative hemorrhage: primary (within 24 hours) and secondary (4-9 days) after surgery | None | None |
| 7. Sibthorpe et al. | 2017 (34) | Australia | Otitis media | Not described | None | 1. Number of patients aged 0–3 years who are Indigenous who received otoscopy at least twice in the last 12 months  b. Number of patients aged 4–6 years who are Indigenous who received otoscopy at least once in the last 12 months  2. Number and proportion of clients aged 0–3 years who are Indigenous who received tympanometry or pneumatic otoscopy at least twice in the last 12 months.  b. Number and proportion of regular clients aged 4–6 years who are Indigenous who received tympanometry or pneumatic otoscopy at least once in the last 12 months  3. Number and proportion of regular  clients aged 0–14 years who are Indigenous with an episode of acute otitis media with or without perforation recorded in the previous 12 months  b. Number and proportion of regular clients aged 0–14 years who are Indigenous with an episode of otitis media with effusion recorded in the previous 12 months  c. Number and proportion of regular clients aged 0–14 years who are Indigenous with an episode of chronic suppurative otitis media or persistent dry perforation recorded in the previous 12 months  4. Number and proportion of episodes of  acute otitis media with or without perforation among regular clients  0–2 years who are Indigenous recorded in the previous 12 months for which an appropriate oral antibiotic was prescribed at time of diagnosis  5. Number and proportion of episodes of  chronic suppurative otitis media among regular clients aged 0–14 years who are Indigenous recorded in the previous 12 months for which an appropriate topical antibiotic was prescribed at time of diagnosis  6. Number and proportion of regular clients  aged 1–14 years who are Indigenous with an episode of recurrent otitis media or persistent otitis media with effusion or persistent chronic suppurative otitis media or persistent dry perforation recorded in the previous 12 months who have a record of audiological testing following diagnosis  7. Number and proportion of regular clients  aged 0–14 years who are Indigenous with an episode of persistent chronic suppurative otitis media or persistent dry perforation or conductive hearing loss >25 dB recorded in the previous 12 months who have a current care plan recorded  8. Number and proportion of episodes of  acute otitis media with or without perforated tympanic membrane recorded in the previous 12 months among regular clients aged 0–14 years who are Indigenous for which a clinical encounter was recorded within 14 days of diagnosis. | None | None |
| Rhinology and skull base surgery | | | | | | | | |
| 1. Cottrell et al. | 2018 (17) | Canada | Chronic rhinosinusitis | Delphi | None | 1. Clinical diagnosis with ≥ one endoscopy or CT finding  2. Differentiation made between CRSwNP and CRSsNP  3. The preferred means of radiological imagining modality of the sinuses in CRS is the CT  4. CRSwNP should be initially managed with topical INCS and short course of oral steroids  5. Clinicians should not prescribe topical or systemic antifungal for CRS  6. Many adjunct therapies commonly used in CRS have limited evidence to support their use. Saline irrigation is an approach that has consistent evidence of benefiting symptoms of CRS  7. Surgery may be beneficial and indicated for individuals with CRS failing appropriate medical treatment  8. Continued use of medical therapy post-surgery is key to success and should be considered for all patients  9. Intravenous and topical antibiotics should not be used for routine CRS | None | None |
| 2. Cottrell et al. | 2020 (19) | Canada | Acute bacterial rhinosinusitis (ABRS) | RAND-UCLA | None | 1. Accurate clinical diagnosis of ABRS is made using signs and symptoms  2. Nasal culture is not required for diagnosis of ABRS  3. Uncomplicated ABRS does not require radiographic imaging  4. Antibiotics may be prescribed for ABRS if disease severely impacts AOL/productivity, the condition worsens, the patient is unable to follow-up, and/or the patient’s condition fails to improve by 7 days after ABRS diagnosis  5. CT scan should be obtained for patients with recurrent ABRS  6. Amoxicillin for 5–10 days should be used as first-line antibiotic therapy for ABRS  7. Adjunct therapy should be prescribed in individuals with ABRS | None | None |
| 3. Hopkins et al. | 2018 (26) | UK | Chronic rhinosinusitis | e-Delphi | None | None | 1. Ability to perform normal activities  2. Acceptability of treatment  3. Compliance with treatment  4. Disease-specific quality of life  5. Endoscopic findings, duration of symptoms  6. Duration of treatment effect  7. Frequency of symptoms  8. Nasal obstruction 9. Need for surgery  10. Overall disease control  11. Overall severity  12. Runny nose  13. Sense of smell  14. Side effects of treatment | None |
| 4. Michel et al. | 2022 (30) | USA | Allergic rhinitis | Not described | None | 1. Sinonasal imaging  2. IgG testing  3. Intranasal corticosteroids or oral antihistamines  4. Leukotriene inhibitors | None | None |
| General otolaryngology–head and neck surgery | | | | | | | | |
| 1. Arce et al. | 2017 (14) | Spain | Inpatient OHNS | Delphi | None | 1. Care delay  2. Surgical management | 1. Complications  2. Mortality  3. Patient complaints  4. Readmissions  5. Safety and satisfaction surveys  6. Average length of stay | 1. Procedure costs  2. Productivity |
| 2. Moraes et al. | 2011 (31) | Brazil | Dysphagia | Not described | None | 1. Swallowing evaluation index  2. Individual care index  3. Speech-language pathology care index  4. Number of assisted patient index  5. Severity rate  6. Diagnosis rate per hospital unit  7. Swallowing rehabilitation demand index  8. Time until first swallowing evaluation  9. SLP per hospital bed index | 1. Time until removal of feeding tube  2. Time until reintroduction of oral feeding  3. Time until decannulation | None |
| Facial plastics and reconstructive surgery | | | | | | | | |
| 1. Manahan et al. | 2020 (28) | USA | Rhinoplasty | Not described | None | 1. Presurgical Discussion of Motivations and Outcomes for Patients Undergoing Rhinoplasty  2. Airway Assessment for Patients Undergoing Rhinoplasty  3. Shared-Decision Making for Postoperative Management of Discomfort following Rhinoplasty | 1. Patient Satisfaction with Rhinoplasty Procedure | None |
