# Supplementary material for: Quality Indicators in Otolaryngology–Head and Neck Surgery: A Scoping Review
Source: J Otolaryngol Head Neck Surg. 2025 Apr 25;54:19160216251330627. doi: 10.1177/19160216251330627 (PMC12035303; doi:10.1177/19160216251330627)
Supplement: sj-docx-2-ohn-10.1177_19160216251330627 – Supplemental material for Quality Indicators in Otolaryngology–Head and Neck Surgery: A Scoping Review [file sj-docx-2-ohn-10.1177_19160216251330627.docx]

| **Table S2.** Study quality appraisal of quality indicator development studies (N = 25) | | | | | | | |
| --- | --- | --- | --- | --- | --- | --- | --- |
| **Author** | **Year** | **Country** | **OHNS subdiscipline** | **AIRE quality appraisal** | | | |
|  |  |  |  | **Purpose, relevance, organization** | **Stakeholder involvement** | **Scientific evidence** | **Additional evidence, formulation, usage** |
|  | | | | Score (%) | Score (%) | Score (%) | Score (%) |
| 1. Arce et al. (14) | 2017 | Spain | General OHNS | 27 | 5.5 | 11 | 15 |
| 1. Balakrishnan et al. (15) | 2015 | USA | Pediatric OHNS | 54 | 44 | 11 | 30 |
| 1. Balakrishnan et al. (16) | 2018 | USA | Pediatric OHNS | 44 | 44 | 11 | 30 |
| 1. Cottrell et al. (17) | 2018 | Canada | Rhinology and skull base surgery | 67 | 61 | 89 | 56 |
| 1. Cottrell et al. (19) | 2020 | Canada | Rhinology and skull base surgery | 67 | 67 | 73 | 61 |
| 1. Cottrell et al. (20) | 2021 | Canada | Otology and neurotology | 80 | 67 | 73 | 63 |
| 1. Cottrell et al. (38) | 2021 | Canada | Otology and neurotology | 73 | 67 | 78 | 61 |
| 1. Cottrell et al. (18) | 2020 | Canada | Pediatric OHNS | 73 | 73 | 78 | 61 |
| 1. Cottrell et al. (21) | 2024 | Canada | Pediatric OHNS | 80 | 67 | 78 | 61 |
| 1. Hall et al. (22) | 2018 | UK | Otology and neurotology | 73 | 67 | 78 | 22 |
| 1. Harman et al. (23) | 2015 | USA | Pediatric OHNS | 70 | 67 | 78 | 26 |
| 1. Hernandez et al. (24) | 2024 | Spain | Head and neck surgery | 73 | 44 | 58 | 30 |
| 1. Hibbert et al. (25) | 2019 | Australia | Pediatric OHNS | 73 | 17 | 56 | 61 |
| 1. Hopkins et al. (26) | 2018 | UK | Rhinology and skull base surgery | 60 | 73 | 78 | 22 |
| 1. Joaquim et al. (27) | 2023 | Portugal | Head and neck surgery | 53 | 50 | 25 | 30 |
| 1. Manahan et al. (28) | 2020 | USA | Plastics and reconstructive surgery | 53 | 44 | 56 | 44 |
| 1. Manduchi et al. (29) | 2024 | Canada | Head and neck surgery | 73 | 78 | 67 | 74 |
| 1. Michel et al. (30) | 2022 | USA | Otology and neurotology/Rhinology and skull base surgery | 73 | 33 | 50 | 33 |
| 1. Moraes et al. (31) | 2011 | Brazil | General OHNS | 47 | 17 | 6 | 23 |
| 1. Noltes et al. (32) | 2022 | Canada | Head and neck surgery | 87 | 56 | 89 | 59 |
| 1. Ouwens et al. (33) | 2007 | Netherlands | Head and neck surgery | 60 | 67 | 73 | 50 |
| 1. Sibthorpe et al. (34) | 2017 | Australia | Pediatrics OHNS | 80 | 59 | 0 | 54 |
| 1. Ten Tije et al. (35) | 2020 | Netherlands | Otology and neurotology | 80 | 56 | 56 | 15 |
| 1. Van Overveld et al. (36) | 2017 | Netherlands | Head and neck surgery | 37 | 73 | 73 | 30 |
| 1. Vila et al. (37) | 2016 | USA | Otology and neurotology | 40 | 67 | 50 | 35 |
|  | | | **Mean** | 63.8 | 54.5 | 55.8 | 41.8 |
|  | | | **Range** | 27–87 | 5.5–78 | 0–89 | 15–74 |

AIRE, Appraisal of Indicators through Research and Evaluation; OHNS, otolaryngology–head and neck surgery
